# Fluffy-like amphiphilic graphene oxide and its effects on improving the antibacterial activity and thermal outstanding of ethyl cellulose /polyvinyl alcohol hydrogel film

**DOI:** 10.1186/s13065-024-01221-3

**Published:** 2024-06-26

**Authors:** Hebat-Allah S. Tohamy, Hossam M. El-Masry

**Affiliations:** 1 Cellulose and Paper Department, National Research Centre, Cairo, 12622 Egypt; 2 Chemistry of natural and microbial products, Pharmaceutical and drug institute, National Research Centre, Cairo, 12622 Egypt

**Keywords:** Amphiphilic graphene oxide, Packaging, sugarcane bagasse, Graphene oxide, Ethyl cellulose, Hydrogel, Antibacterial activity

## Abstract

The antibacterial characteristics of graphene oxide (GO-SB) nano-sheets generated by charring sugarcane bagasse (SB) are described in this study. The antibacterial capability of GO-SB was improved when it was grafted with ethyl cellulose (EC) and polyvinyl alcohol (PVA) to form GO-SB/EC/PVA hydrogels. Characterization of GO-SB nanosheets and GO-SB/EC/PVA hydrogels was accomplished by using FTIR, SEM, XRD, and thermal studies. The antimicrobial activity was carried out against Gram positive bacteria [*Micrococcus leutus* & *Staphylococcus aureus*], Gram negative bacteria [*Escherichia coli*, *Pseudomonas aeruginosa*] and pathogenic fungal yeast [*Candida albicans*] applying the disc diffusion method. The disc diffusion method results showed that the improved GO-SB/EC/PVA exhibited a reasonable level of antimicrobial capability against *Micrococcus leutus*, demonstrating that the antimicrobial improvement of GO-SB was more effective in the GO-SB/EC/PVA hydrogels by increasing the inhibition zone of Gram-positive bacteria, *Micrococcus leutus* from (13.0 to 16.0 mm).

**Graphical abstract:**

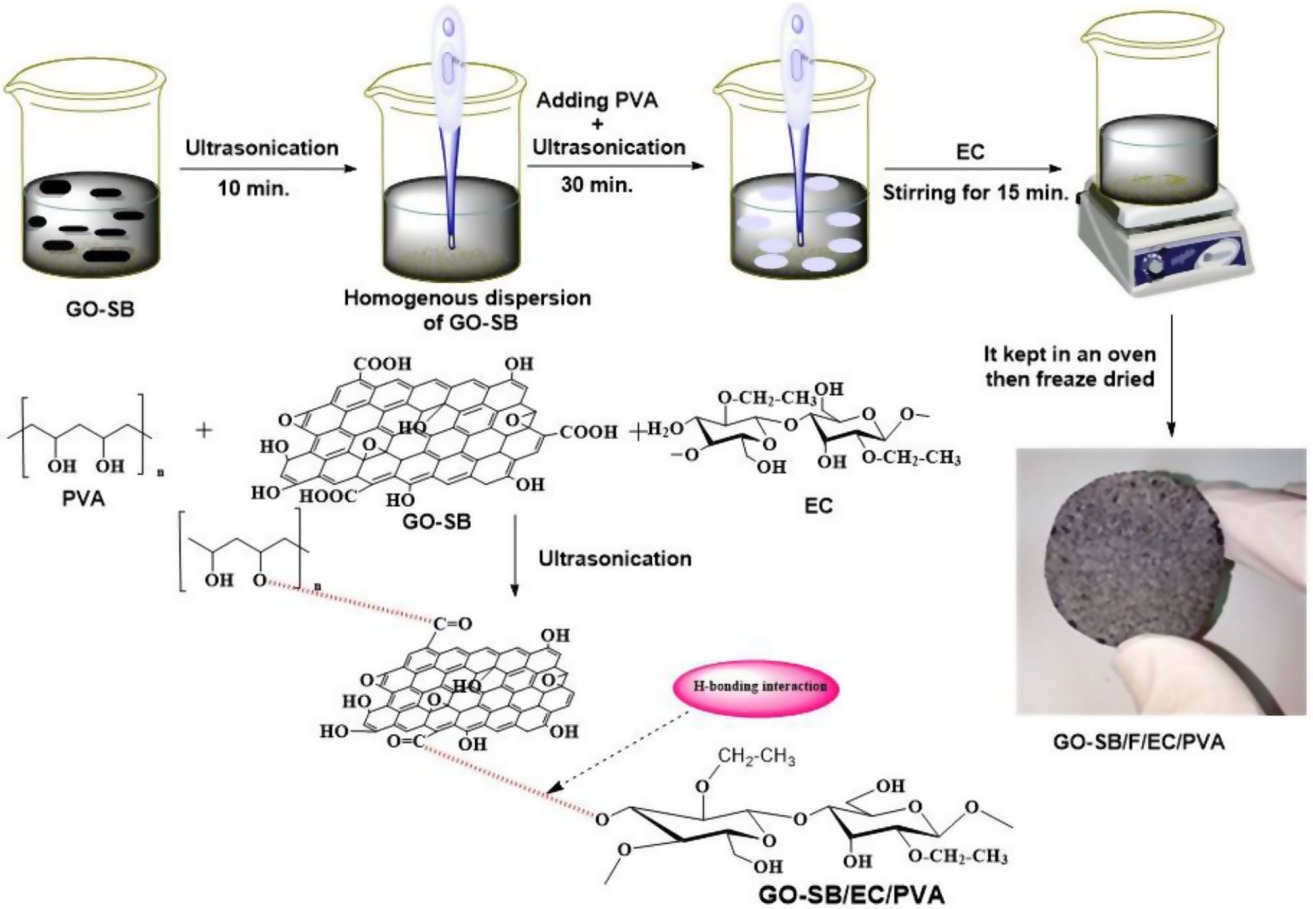

## Introduction

The realm of packaging materials is vast and varied. Metals and glass are two commonly employed options due to their exceptional resistance to corrosion and their inherent strength. However, polymers, particularly plastics, reign supreme in terms of popularity. Their numerous advantages include transparency, affordability, flexibility, heat-sealing capabilities, and an impressive strength-to-weight ratio. Unfortunately, plastics are non-biodegradable, posing a severe global environmental threat. Due to concerns with their performance and processing, such as brittleness and thermal distortion temperature, the use of biodegradable polymers has been limited [1]. To address this issue, several researchers are working to create eco-friendly packaging solutions that take advantage of the ecological benefits of biopolymers in food packaging applications. Hydrogels have the potential to open up new avenues for the creation of efficient biopolymer packaging materials with desirable properties [2, 3]. The shelf life of food cannot be extended by pure ethyl cellulose (EC) film, however adding an antibacterial agent can prevent germs from acting on the film’s surface [4]. In comparison to films, hydrogels may offer new opportunities for the creation of effective biopolymer packaging materials with desirable properties [1, 3]. Due to their ability to absorb water without disintegrating, hydrogels are polymer networks that expand when exposed to water [3, 5, 6]. There are several advantages to using sustainable hydrogels in packaging sector, including their biodegradability, low density, low price, and minimal energy consumption during production [1, 3]. Additionally, the hydrogels’ porous nature enables the controlled release of the majority of antibacterial chemicals. Because of this, hydrogels have developed into superior antibacterial agent carriers. Antimicrobial drugs have been loaded using it frequently in the interim. Consequently, antimicrobial composite hydrogel development has become a popular issue [7].

Poly vinyl alcohol (PVA) is widely grafted with biopolymers such as cellulosic polymer due to its advantageous properties such as gas permeability and biocompatibility [8]. To accelerate antibacterial activity, EC can stimulate the synthesis of extracellular matrix and the activation of macrophages [9]. PVA and EC could preserve swelling ability [10]. However, the spread of bacteria frequently leads to food spoilage [2, 3, 11]. Graphene oxide (GO–SB) will be employed as an antibacterial agent to address this issue. GO-SB has bactericidal properties against all pathogenic microorganisms [2, 12]. GO–SB has emerged as a potential nanomaterial for improving antibacterial capabilities due to its outstanding properties such as high oxygen density, high specific area, chemical resistance, and thermal stability [2, 13, 14]. The GO–SB is highly effective against Gram-positive and Gram-negative bacteria. It could physically or chemically interact with bacterial cells. GO–SB may cause structural damage to the bacterium, resulting in cell death. When GO–SB interacts with bacteria, harmful chemicals are released, resulting in biological death [2, 12].

Under atmospheric conditions, GO–SB might be produced by oxidising sugarcane bagasse agrowastes (SB) [2, 14]. The GO–SB comprises both hydrophilic oxygenated groups and hydrophobic conjugated graphitic-regions (i.e., it is amphiphilic). The amphiphilic characteristic of GO-SB allows it to be dispersed readily in the membrane matrix [15].

We present here the development of eco-friendly GO-SB nanosheets from SB through oxidation in normal ambient conditions, as opposed to Hummer’s approach (which causes major environmental problems). The produced GO–SB was then grafted with EC/PVA copolymers to create a GO-SB/EC/PVA hydrogel film for use as an antibacterial agent.

## Experimental

### Materials

Sugarcane bagasse (SB) was purchased from the Quena Paper Industry Company in Egypt. Sisco laboratories Pvt. Ltd. (India) supplied the ferrocene (FER). All materials and reagents were utilized without undergoing any further purification.

### Preparation of graphene oxide from sugarcane bagasse (GO–SB)

For 10 min, a muffled oven at 300 °C was used to char a combination of 0.5 g SB and 0.1 g FER [2, 14].

### Synthesis of graphene oxide-based EC/PVA hydrogels (GO–SB/EC/PVA)

By ultrasonically dispersing 100 mg of GO-SB in water, a uniform suspension sheet (10 mg/ml) was produced. This GO–SB dispersion was combined with 10 mg/L of PVA aqueous solution and 200 mg EC, and the combination was ultrasonically processed for 30 min. The GO–SB/EC/PVA hydrogel film was prepared by heating the resultant dispersion in an oven at 50 °C [16].

### Raman analysis

spectroscopy using Raman A WITEC Focus Innovations Alpha-300 Raman Confocal Microscope was used for the Raman spectroscopy. The relationship could be used to compute the crystal size:

1 $$Crystal size \left(nm\right)=C \left(\lambda \right)X \left(\frac{\text{I}\text{G}}{ID}\right)$$

where C is the pre factor at 532 nm and equals to 4.7 and I_G_, I_D_ are the intensities of G– and D– bands, respectively [17].

### FTIR analysis

The KBr disc method was used by the Mattson 5000 spectrometer (Unicam, United Kingdom) for FTIR analysis.

### XRD analysis

As the diffraction patterns were examined using a Bruker D-8 Advance X-ray diffractometer (Germany)using a 40 kV voltage and a 40 mA current while using copper (K) radiation (1.5406), the crystallinity (Cr. I) was investigated. The following formulas can be used to determine the Cr. I. (%)d-spacing (thickness):

2 $$Cr.I. \left(\%\right)=\frac{\text{S}\text{c}}{St} X 100$$

3 $$Thickness \left(d, nm\right)=\frac{{\uplambda }}{2 sin\theta }$$

where Sc = area of the crystalline domain, St = area of the total domain, θ = Bragg’s angle in radians and λ = X-ray wavelength (0.1542 nm), respectively [18–20].

### Morphological properties

Using a transmission electron microscope (TEM, JEOL JEM-2100) and scanning electron microscopy (SEM, Quanta-250) at an acceleration voltage of 120 kV, the morphology of the materials was examined.

### Thermal properties

A Perkin Elmer thermogravimetric analyzer was used to conduct the TGA examination at 1000 °C and a rate of 10 °C/min in a N_2_ environment. Using the Coats-Redfern method, the thermal degradation kinetics of the prepared R1, R2, and R3 samples were investigated:

4 $$\eqalign{ Log\left[ {{{1 - {{\left( {1 - \alpha } \right)}^{1 - n}}} \over {{T^2}(1 - n)}}} \right] = & \cr & log{{AR} \over {\beta E}}\left[ {1 - {{2RT} \over E}} \right] - {E \over {2.303RT}}\,for\,n\, \ne \,1 \cr}$$

5 $$\eqalign{ Log\left[ {{{ - log(1 - \alpha )} \over {{T^2}}}} \right]\, = & \cr & log{{AR} \over {\beta E}}\left[ {1 - {{2RT} \over E}} \right] - {E \over {2.303RT}}\,for\,n\, = \,1 \cr}$$

where n is the order of the degradation reaction, denotes the fractional conversion, ß (K/min) denotes the heating rate, T (K) denotes the temperature, R (kJ/mol.K) denotes the gas constant, A (s^− 1^) denotes the frequency factor, and E denotes the activation energy [5, 19, 21]. Equation (6) was used to estimate the other kinetic parameters, such as enthalpy (ΔH), entropy (ΔS), and free energy change (ΔG).

6 $$\eqalign{& \Delta {H^*}\, = \,{E^*}\, - \,RT, \cr & \Delta {G^*}\, = \,\Delta {H^*}\, - T\Delta {S^*}\,{\rm{and}} \cr & \Delta {S^*} = 2.303\left( {\log {{Ah} \over {KT}}} \right)R \cr}$$

where (h) and (k) are Planck and Boltzman constants [2, 6].

### Swelling capacity

At 37 °C for one day, the starting weight of GO-SB/EC/PVA (W_i_) was incubated while being immersed in 20 ml of PBS solution (pH 7.4). After a day, the GO-SB/EC/PVA was taken out of the solution. Tissue paper was used to wipe the composite surface of the sample clean of the PBS solution. The wet weight (W_f_) of GO-SB/EC/PVA was then calculated. The swelling percentage (S %) for GO-SB/EC/PVA was computed using the following equation:

7 $$S \left(\%\right)=\frac{\text{W}\text{f}-\text{W}\text{i}}{Wi} X 100$$

where W_f_ and W_i_ are the mass of the swollen gel initially and finally, respectively [6].

### Antimicrobial assay

The antimicrobial test was carried out via the disc diffusion method according to previous work [11].

## Results and discussion

**Fig. 1:**
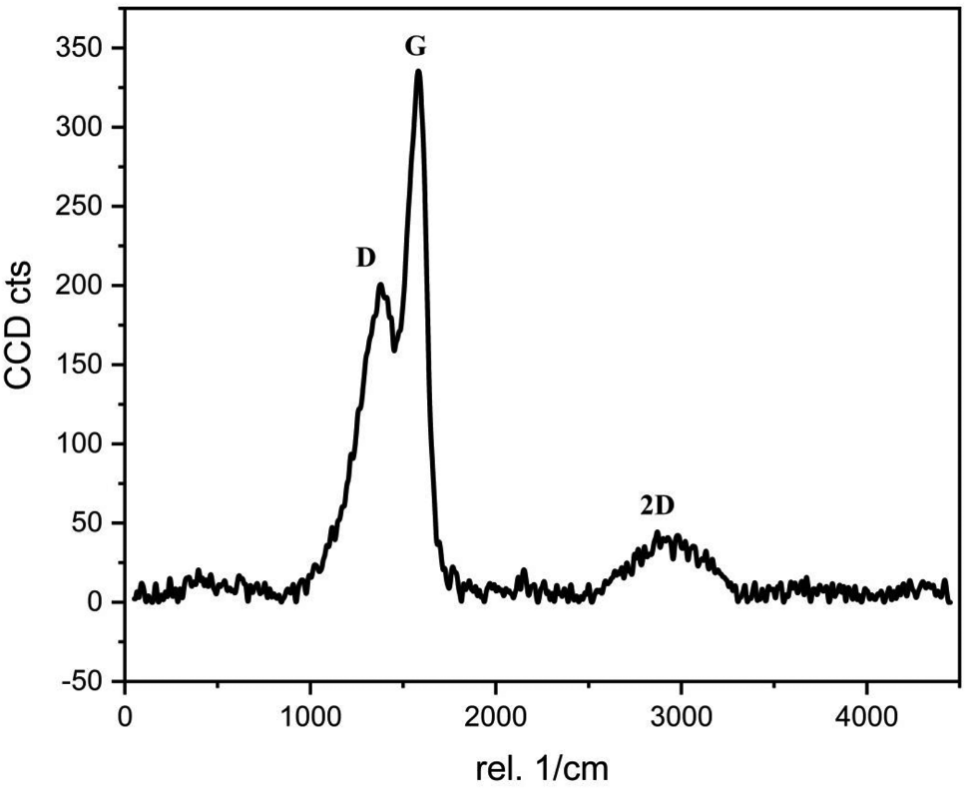
Raman spectra of GO–SB

The calculated crystal size for GO–SB was 7.89 nm. The prepared GO–SB’s Raman spectra are displayed in Fig. 1. The D–band, which occurs as a result of disorder in the atomic arrangement, is positioned at 1583.50 cm^− 1^, while the G–bands, which result from the stretching of the sp^2^ carbon, are placed at 1375.00 cm^− 1^ [19]. I_D_/I_G_ peak intensity ratios were 0.59. At 2874.50 cm^− 1^, the 2D–band is situated which used to determine how many layers there are in the resulting GO–SB. In the case of single layer G, the 2D–band is situated at approximately 2679 cm^− 1^. For multilayer G (2–4 layers), the location moves by about 19 cm^− 1^. These allow us to infer that the GO–SB is multilayered and has been prepared. Additionally, for single-, double-, triple-, and multi-layer G (> 4), the I_2D_/I_G_ intensity ratios are frequently less than 1.6, 0.8, 0.30, and 0.07, respectively. The preparation of multilayered (less than 4) sheets is required since the calculated I_2D_/I_G_ ratio is less than 0.12 [14].

**Fig. 2:**
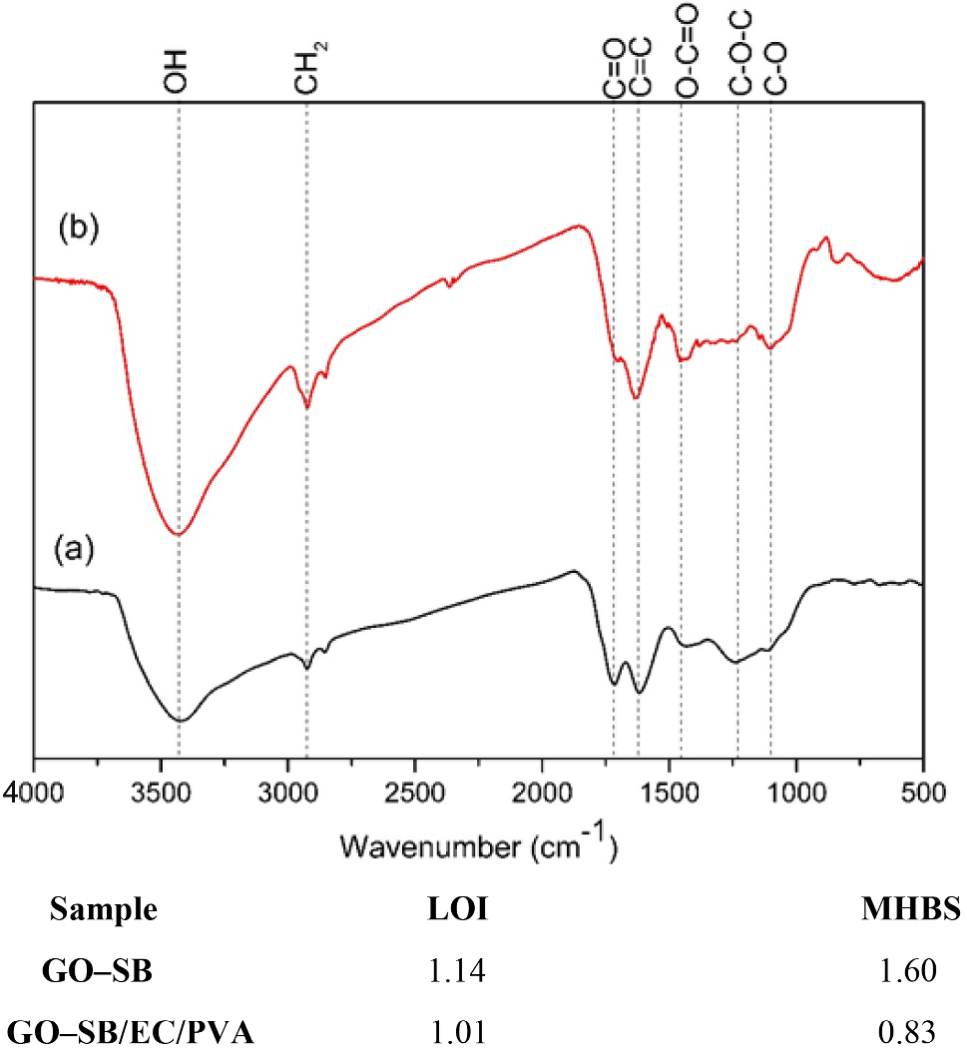
FTIR spectra of GO–SB and GO–SB/EC/PVA

The S (%) of GO–SB/EC/PVA was 285%. Figure 2 depicts the FTIR spectra of the synthesised GO–SB and GO–SB/EC/PVA. Both GO–SB and GO-SB/EC/PVA have peaks between 3439 and 3441 (O–H), 1708–1722 (C = O), 1627–1647 (C = C), 1439–144 9 (O-C = O), 1094–1244 (C–O), and 1228–1232 cm^− 1^ (C–O–C). The FT-IR spectra of GO–SB revealed the presence of oxygenated groups as a result of SB oxidation. The bands at 2921–2935 cm^− 1^ and 2852–2866 cm^− 1^ are assumed to be caused by the asymmetric and symmetric forms of CH_2_ [2, 14]. The addition of PVA is confirmed by the additional peak at 2369 cm^− 1^ in the GO–SB/EC/PVA, which is the normal absorption peak of C = C = C [16].

The O–H group’s relative intensity (RA) was at 3.0 and 0.83, whereas the C = O group’s RA was at 14.70 and 1.04 for GO–SB and GO–SB/EC/PVA, respectively. This demonstrates that the free O–H groups of the GO–SB have been cross-linked with PVA groupings. For GO–SB and GO–SB/EC/PVA, the RA of the C = C is 0.75 and 1.007, respectively. After modification with EC/PVA, it was discovered that the O–H stretching for GO–SB (3441 cm^− 1^) shifted to a lower frequency (3439 cm^− 1^) for GO-SB/EC/PVA. This adjustment may have caused a decrease in intermolecular H–bond formation, which was observed to occur [2, 14, 22].

The empirical crystallinity index (LOI) and mean H–bond strength (MHBS) values were decreased as a result of grafting EC/PVA into the GO–SB backbone. Figure 1 displays the LOI and MHBS values [11, 14, 19, 22].

Furthermore, the C = O group (1722 cm^− 1^) in GO–SB has the potential to make bonds with the functional groups on EC/PVA. As a result, for GO–SB/EC/PVA, the C = O stretching frequency is moved to a lower wavenumber value at 1708 cm^− 1^ [2, 11, 22].

**Fig. 3:**
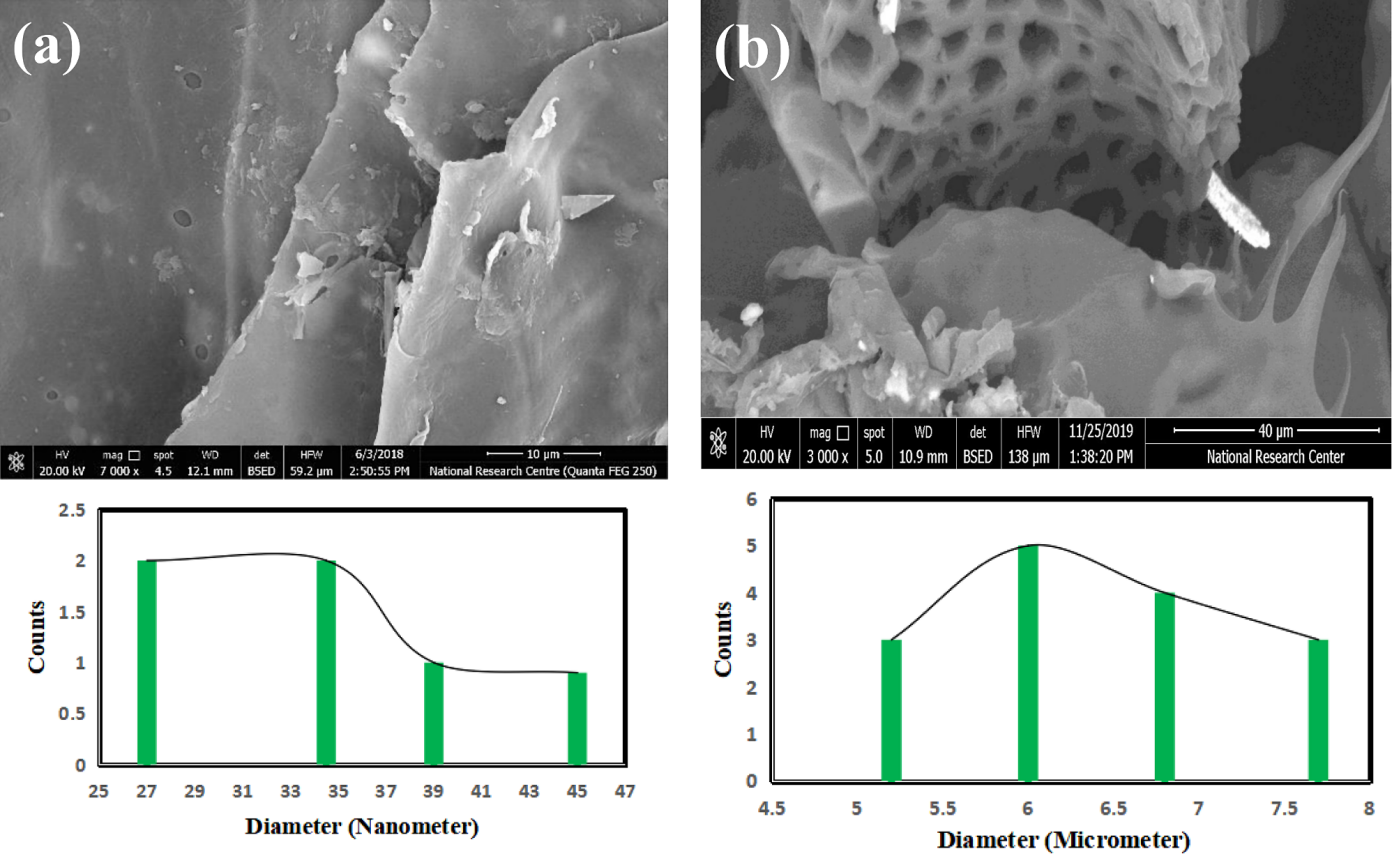
SEM and pore size distribution images of (**a**) GO–SB and (**b**) GO–SB/EC/PVA

Images taken using SEM of the generated GO–SB nanosheets and GO–SB/EC/PVA hydrogel are shown in Fig. 3. With pores of 34.84 nm in size, the morphology of GO–SB showed wrinkled, fluffy sheets. These fluffy wrinkles were attributed to oxidation-induced disruption of the planar sp2-hybridized carbon [2, 14, 18]. The GO–SB/EC/PVA displayed a micro-sized cylindrical honey comb structure with a pore size of around 6.00 $\mu$m that was embedded between GO–SB/EC/PVA hydrogel, as illustrated in Fig. 3b. According to SEM images, GO–SB/EC/PVA hydrogel showed enhanced porosity after EC/PVA was grafted onto GO–SB surface.

XRD showed two peaks in the GO–SB at 2 = 9.30° (001), which is a typical signal for GO–SB, and 2 = 21.80° (002) [2, 14, 18]. The XRD peaks of GO–SB are in tune with JCPDS data (89-7213) [23].

**Fig. 4:**
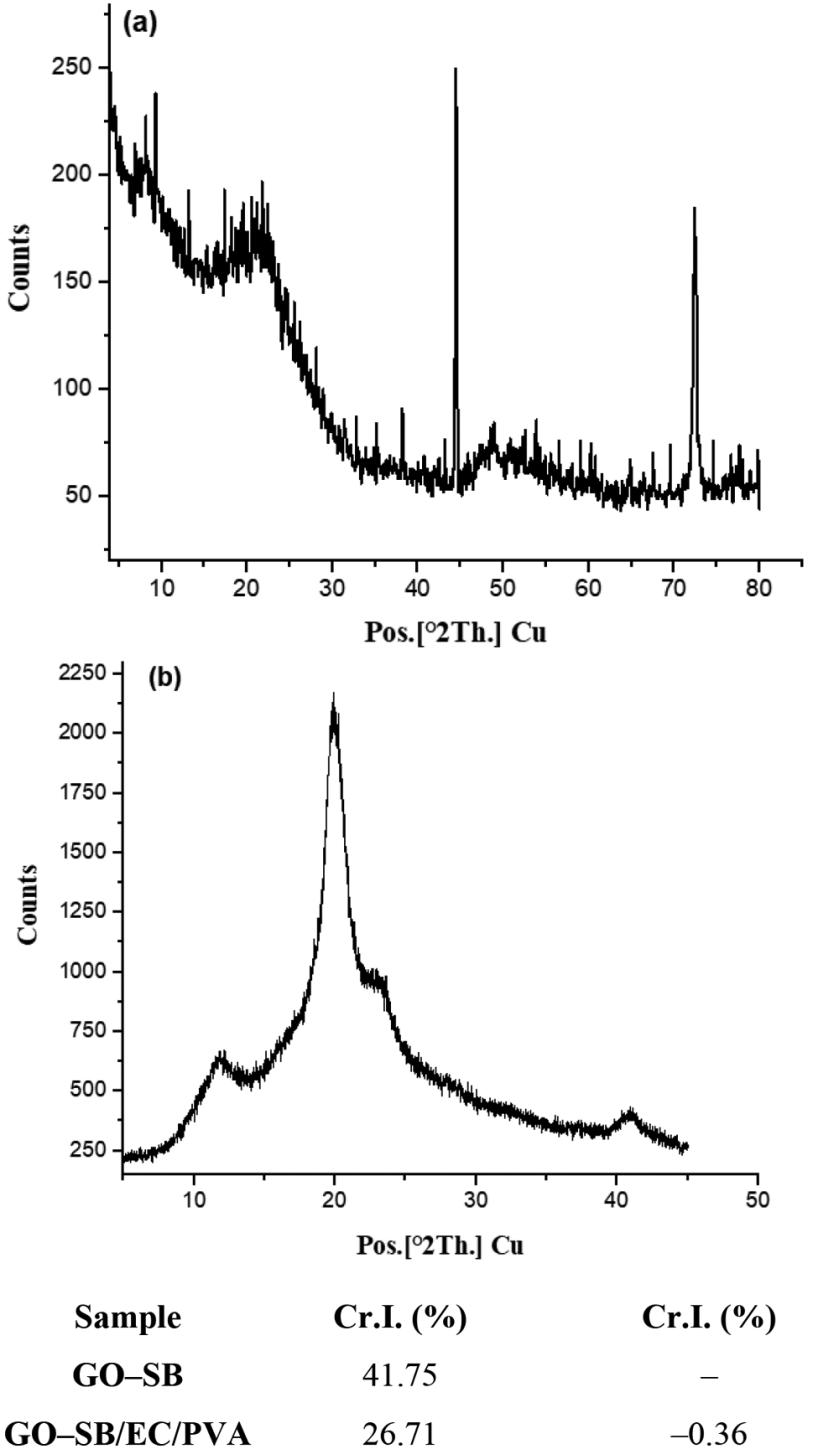
XRD of (**a**) GO–SB and (**b**) GO–SB/EC/PVA

In the GO–SB/EC/PVA pattern shown in Fig. 4, there are four weak diffraction peaks at 2θ = 11.5, 19.8, 23.2, and 40.9°. Two faint diffraction peaks at 2θ = 11.5° and 23.2° may be caused by grafting on GO–SB lattice, and the diffraction peak at 2θ = 19.8° should be attributed to the distinctive diffraction peak of chain of PVA. The faint peak at 2 = 40.9° could be the GO–SB lattice’s distinctive diffraction peak [16].

The Cr.I (%) of GO–SB/EC/PVA was found to decrease compared to that of GO–SB due to grafting which cleaved H–bonds [2, 14, 18]. The XRD Cr.I. (%) was compatible with the MHBS values determined by FTIIR spectra.

**Fig. 5:**
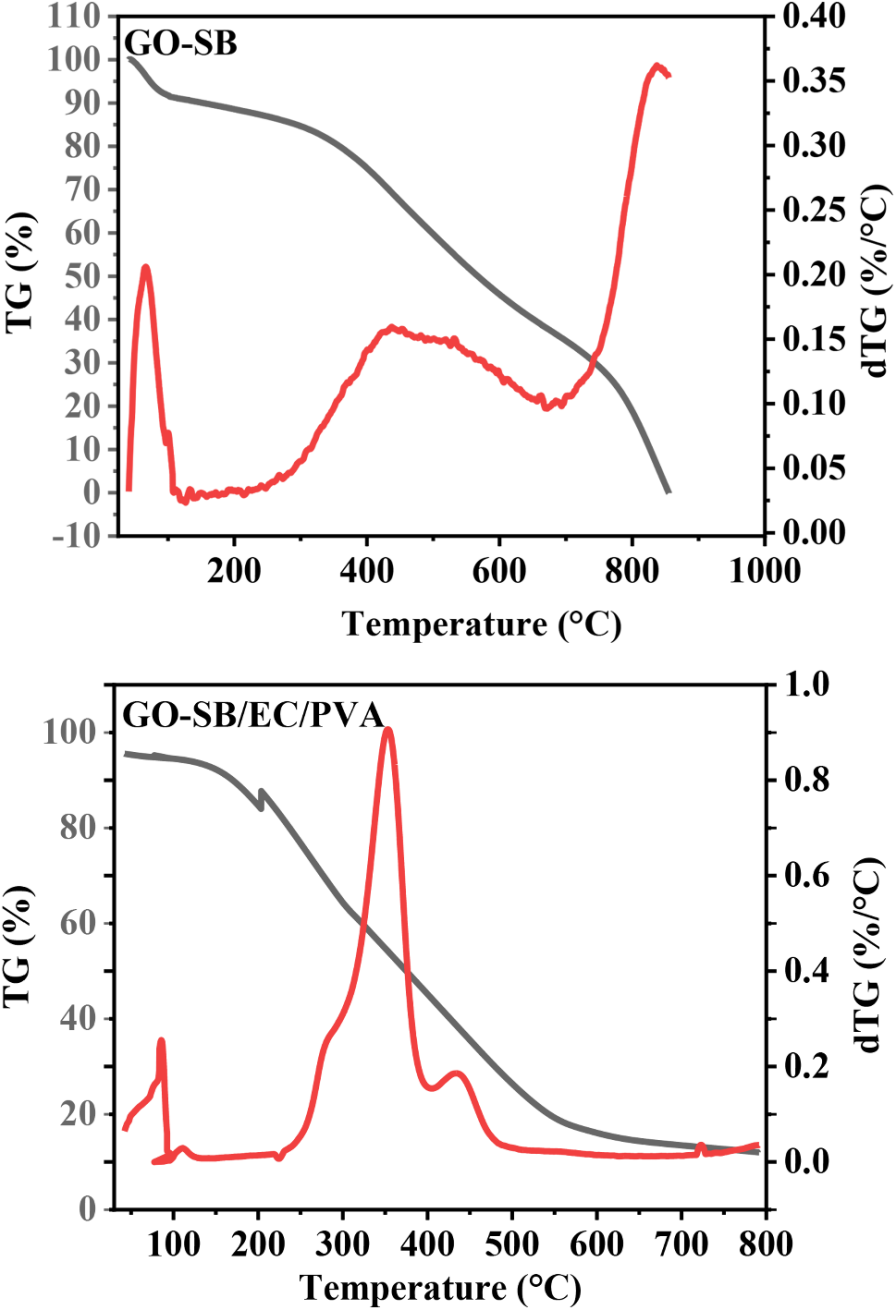
TGA/DTG of EC, GO–SB and GO–SB/EC/PVA

Indicating the presence of some non-volatile components, the TGA/DTG of EC, GO–SB, and GO–SB/EC/PVA revealed weight losses of 75.08, 76.80, and 85.00%, respectively, at 1000 °C (Fig. 5; Table 1) [5].

**Table 1:** TGA/DTG data of EC, GO–SB and GO–SB/EC/PVA

Sample	Weight loss at 1000 °C, %					Residual weight, %	Temp. of steps in TGA, °C		
	Step 1	Step 2	Step 3	Step 4	Total loss, %		Step 1	Step 2	Step 3
EC	3.83	71.25	–	–	75.08	24.92	196.69	346.32	–
GO–SB	13.20	44.18	19.42	–	76.80	23.2	64.64	431.72	836.32
GO–SB/EC/PVA	3.77	60.00	17.73	3.50	85.00	18.50	110.71	352.70	439.58

For EC, the weight loss (WL) ranged between 38.61 and 238.79 °C, with a maximum at 196.69 °C (average WL of 3.83%), which was most likely brought on by dehydration during the thermal degradation of EC. The combustion produced the 2nd WL, which ranged between 262.7 and 425.82 °C with a high temperature of 346.32 °C (average WL ≈ 71.25%) [5, 6].

For GO–SB, the 1st WL occurred between 45.10 and 252.73 °C, with a maximum at 64.64 °C (average WL of 13.20 and 3.77%, respectively), and was probably brought on by dehydration. The thermal degradation mechanisms of GO–SB could be separated into three reaction phases. Pyrolytic fragmentation produced the second WL, which ranged between 252.86 and 627.07 °C with a maximum at 431.72 °C (average WL of 44.18%). The carbonization-related 3rd decomposition process had temperatures ranging between 647.29 and 855.19 °C, with a maximum at 836.32 °C (average WL of 19.42%) [6, 19].

The thermal decomposition processes of GO–SB/EC/PVA could be divided into four major reaction steps, where the 1st WL was between 42.33 and 126.31 °C, with a maximum at 110.71 °C (average WL of 3.77%) was likely caused by dehydration. The 2nd WL was between 303.39 and 406.70 °C, with a maximum at 352.70 °C (average WL of 60%) was the result of dehydroxylation and pyrolytic fragmentation. The 3rd decomposition step between 413.95 and 791.33 °C, with a maximum at 439.58 (average WL of 17.73%), which was related to the carbonization [2, 6, 19].

The molecular structure and bonding energy of EC are altered during its transformation into GO–SB/EC/PVA hydrogel, which results in a difference in the thermal behavior between EC and GO-SB/EC/PVA (Tables 2, 3).

**Table 2:** Thermo analytical and thermodynamic data of the thermal decomposition steps of the EC, GO–SB and GO–SB /EC/PVA

Sample	Stage	TGA range, °C	DTA peak, °C	*n*	*R* ^2^	A (s^− 1^)	ΔH (kJ mol^− 1^)	Δs (kJ mol^− 1^)	ΔG (kJ mol^− 1^)	SE	E, (kJ mol^− 1^)
EC	1st	38.61–238.79	196.69	1.5	0.944	0.38	6.51	–0.25	127.05	42 × 10^− 2^	10.42
	2nd	262.76–425.82	346.32	1.5	0.911	0.30	17.78	–0.26 **∑∆S= − 0.51**	179.37 **∑∆G = 306.42**	14 × 10^− 1^	22.93 **∑E = 22.93**
GO–SB	1st	45.11–252.73	64.64	0	0.999	19.53	–0.06	–0.22	71.35	75 × 10^− 4^	2.63
	2nd	252.86–627.07	431.72	1.5	0.995	14.33	17.30	–0.22	89.55	16 × 10^− 2^	19.99
	3rd	647.29–855.19	836.32	1.5	0.995	15.17	17.71	–0.25 **∑∆S= − 0.69**	204.68 **∑∆G = 365.58**	46 × 10^− 3^	24.45 **∑E = 44.44**
GO–SB/EC/PVA	1st	42.33–126.31	110.71	0	0.999	1.00	–3.19	–0.24	91.60	3.93 × 10^− 8^	11 × 10^− 4^
	2nd	303.39–406.70	352.70	0	0.999	1.00	–5.20	–0.25	151.92	6.05 × 10^− 9^	6.13 × 10^− 5^
	3rd	413.95–557.00	439.58	0	0.999	2.45	6.53	–0.24	180.91	53 × 10^− 4^	12.45
	4th	607.01–791.33	723.27	1.5	0.999	3.29	6.32	–0.24 **∑∆S= − 0.97**	250.48 **∑∆G = 674.91**	20 × 10^− 4^	16.06 **∑E = 27.05**

**Table 3:** Inhibition zone diameter (millimeter) of the samples

Test bacteria		Samples		
		Treated sample		
		Sample (1) GO–SB /EC/PVA	Sample (2) GO–SB	CN**
**1-**	***Escherichia coli***	Nil*	Nil	20.0
**2-**	***Pseudomonas aeruginosa***	Nil	Nil	13.0
**3-**	***Micrococcus leutus***	16.0	13.0	23.0
**4-**	***Staphyllococus aureus***	Nil	Nil	21.0
**5-**	***Candida albicans***	Nil	Nil	ـــــــــــــــ

*Nil: No antimicrobial activity was recorded

**CN.: Gentamicin 10 µg

This recommendation is supported by the following evidence:

- The rise in computed activation energy (E_a_) for GO–SB/EC/PVA from 22.93 kJ mol^− 1^ for EC to 27.05 22.93 kJ mol^− 1^ for GO–SB/EC/PVA demonstrates the thermal superiority of GO–SB/EC/PVA over EC/PVA film owing to grafting with GO–SB [2, 6, 19].
- The ΔS for EC, GO–SB, and GO–SB/EC/PVA are all negative, suggesting that the combustion was not spontaneous. The higher ΔS (-0.97 kJ mol^− 1^) of GO–SB/EC/PVA suggests that its thermal degradation was more complex and required more external energy to deteriorate than EC (-0.51 kJ mol^− 1^) and GO–SB (–0.69 kJ mol^− 1^) [2].
- The value of ∑∆G for GO–SB/EC/PVA (674.91 kJ mol^− 1^) is higher than EC (306.42 kJ mol^− 1^) and GO–SB (365.58 kJ mol^− 1^), indicating the GO–SB/EC/PVA is more non-spontaneous and require external heat input than the EC and GO–SB, which improves their high thermal stability [21].
- The values of H for EC, GO–SB and GO–SB/EC/PVA are low in the 1st stage and start to be increased in the final stage (i.e. from 6.51 to 17.78 kJ mol^− 1^ for EC, from − 0.06 to 17.71 kJ mol^− 1^ for GO–SB and from − 3.19 to 6.32 kJ mol^− 1^ for GO–SB/EC/PVA), indicating the very high energy requirement as the reaction progresses [1, 21].
- The pre-exponential factor (A) describes the relationship between the temperature and the reaction rate constant. The A values for GO–SB/EC/PVA are increased from 1.00 to 3.29 s^− 1^ which meaning their complex structure [25].

**Fig. 6:**
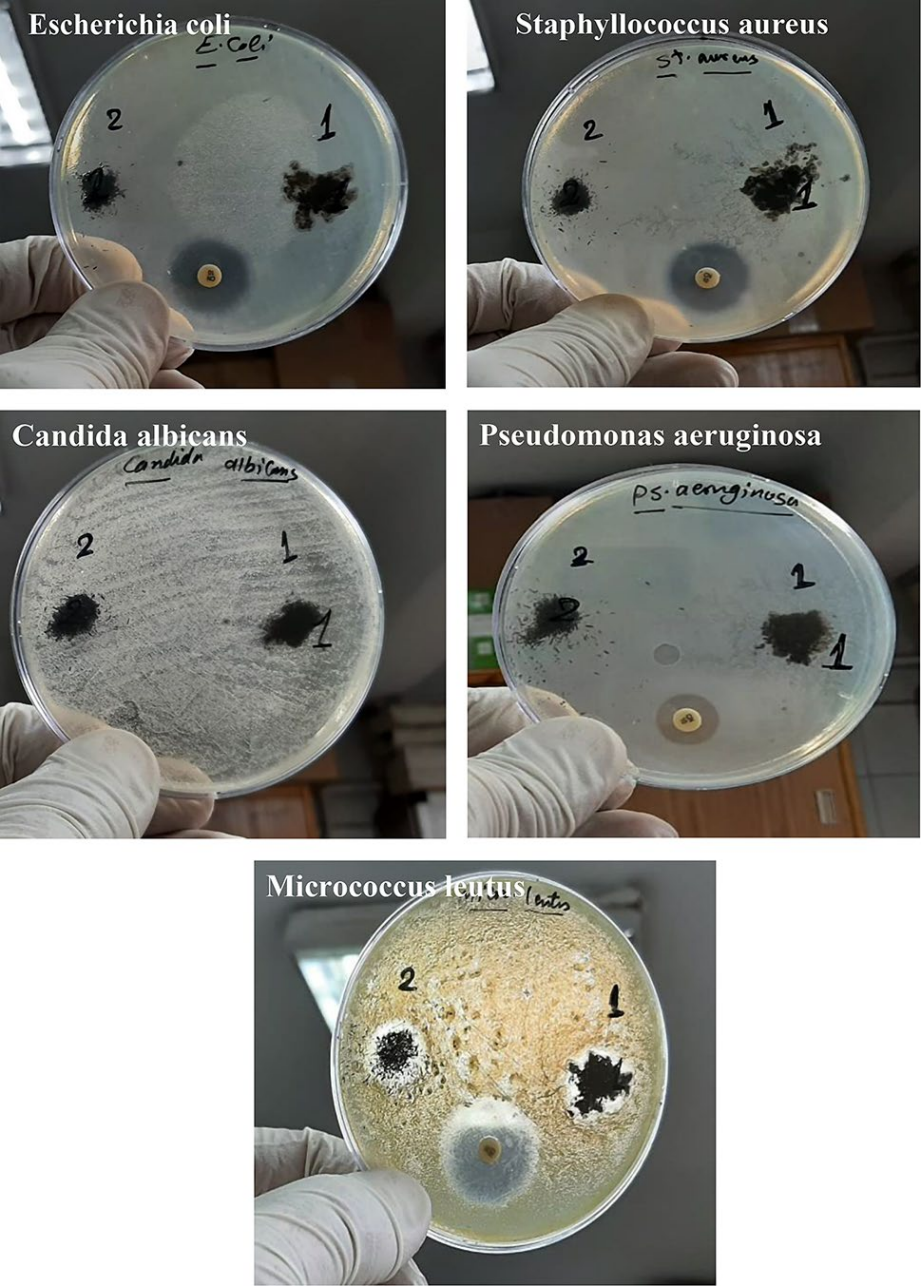
Antimicrobial activity of the prepared samples. 1-GO–SB/EC/PVA. 2-GO–SB

The findings revealed that GO–SB and GO–SB/EC/PVA exhibited no antibacterial or antifungal efficacy against pathogenic gram-negative bacteria (such as *E. coli* and *Pseudomonas aeruginosa*) or fungal yeast (such as *Candida albicans*). Furthermore, GO–SB and GO–SB/EC/PVA demonstrated no antibacterial activity against gram-positive bacteria (e.g., *Staphylococcus aureus*) (Fig. 6). Because their cell membranes are resistant to disintegration or destruction. Therefore, none of these bacterial strains demonstrate any response. 

GO–SB and GO–SB/EC/PVA exhibit potent antibacterial activity against gram-positive bacteria such as *Micrococcus leutus*. This is evidenced by their specific antibactericidal spectrum, with inhibition zones ranging from 13 to 16 mm. This promising anti-gram-positive bacterial activity is likely due to the ability of oxygen groups in GO–SB can interact with lipids, proteins, and DNA/RNA of bacterial cell via H-bonds, π-π, and electrostatic bonds [24–26].

## Conclusion

The oxidation of SB in muted conditions resulted in the successful production of GO–SB. To improve the antibacterial properties of the hydrogel, the eco-friendly GO–SB was grafted with EC and PVA. The GO–SB/EC/PVA showed the greatest susceptibility to *Micrococcus leutus*, with a peak inhibition zone of 16.0 mm. Moreover, GO-SB/EC/PVA outperformed GO–SB in antibacterial efficacy against the studied strains of *Micrococcus leutus*, increasing antimicrobial activity while extending the inhibition zone from 13.0 mm to 16.0 mm. FTIR, SEM, XRD, and TGA/DTG analyses have been used to characterise GO–SB and GO–SB/EC/PVA. The results demonstrated that EC/PVA had an outstanding effect on the GO–SB/EC/PVA. It improves the GO-SB/EC/PVA hydrogel film’s thermal stability. The RA of the O–H group was 3.2 and 0.83, although the intensity of the C = O group was 14.70 and 1.04; whereas the intensity of the C = O group was 14.70 and 1.04 for GO–SB and GO–SB/EC/PVA, respectively. This verifies the substitution of EC/PVA groups for the free OH groups of GO-SB.

## Data Availability

No datasets were generated or analysed during the current study.
